# Congenital Nasolacrimal Duct Obstruction: Natural Course, Diagnosis and Therapeutic Strategies

**DOI:** 10.3390/jcm14113716

**Published:** 2025-05-26

**Authors:** Katarzyna Błaszczyk, Kamil Biedka, Agata Estreicher, Michał Wesołowski, Jakub Bulski, Aleksandra Sobaś, Oliwia Ziobro, Filip Maj, Karol Sornat, Anna Klasa, Jakub Karwacki, Tadeusz Sebzda

**Affiliations:** 1 Department of Physiology and Pathophysiology, Division of Pathophysiology, Wroclaw Medical University, 50-368 Wroclaw, Poland; blaszczykrzyk@gmail.com (K.B.); agataestreicher@gmail.com (A.E.); michal.wesolowski@umw.edu.pl (M.W.); oziobro@gmail.com (O.Z.); jakub.karwacki@umw.edu.pl (J.K.); tadeusz.sebzda@umw.edu.pl (T.S.); 2 Faculty of Medicine, Collegium Medicum, Jan Kochanowski University, 25-317 Kielce, Poland; jakub.bulski@icloud.com (J.B.); aleksandra.ewasobas@gmail.com (A.S.); filip.jacek.maj@gmail.com (F.M.); karol.b.sornat@gmail.com (K.S.); 3 University Hospital in Kraków, 30-688 Kraków, Poland; klasa.annamaria@gmail.com; 4 University Center of Excellence in Urology, Department of Minimally Invasive and Robotic Urology, Wroclaw Medical University, 50-556 Wroclaw, Poland

**Keywords:** congenital nasolacrimal duct obstruction, nasolacrimal duct, epiphora

## Abstract

Congenital nasolacrimal duct obstruction (CNLDO) is a prevalent condition in newborns, characterized by lacrimation excess due to anatomic occlusion of the nasolacrimal ducts (NLD). This narrative review provides a comprehensive overview of CNLDO, encompassing its etiology, epidemiology, clinical manifestations, diagnosis and current treatment options. Diagnosis comprises the fluorescein dye disappearance test (FDDT) and imaging studies. In most cases, CNLDO resolves spontaneously within the first year of life. However, if spontaneous resolution does not occur, or if complications arise during observation period, treatment with conservative methods such as massage is introduced earlier, depending on the severity and complexity of the case. If these methods prove unsuccessful, more invasive procedures, including probing, intubation or dacryocystorhinostomy, are employed. While early diagnosis is crucial, its direct impact on treatment outcomes is more related to the prompt identification of cases that may require escalation of care.

## 1. Introduction

Congenital nasolacrimal duct obstruction (CNLDO) is defined as an anatomical abnormality of the lacrimal organ. This condition is characterized by the presence of excessive tears, medically termed ‘epiphora’ [1]. The lacrimal apparatus is a complex system that plays an important role in maintaining tear film stability, corneal clarity, and ocular surface health. The tear system is composed of two primary components: the glands that secrete tears and the drainage system, which is responsible for their efflux. The tear drainage system comprises upper and lower tear points, tear ducts, tear sacs, and nasolacrimal ducts (NLD) [2,3]. Converging to form a unified pathway, these components facilitate the drainage of tears into the lower nasal duct within the nasal cavity. Beyond its function as the primary drainage system, this system also modulates the flow of tears and contributes to the maintenance of the equilibrium of the tear film [4,5]. The aim of this narrative review is to provide a comprehensive and up-to-date synthesis of current knowledge on CNLDO. The article focuses on describing the embryological development and pathophysiological mechanisms underlying the condition, presenting current epidemiological data and risk factors, and outlining the clinical manifestations and diagnostic methods, with particular emphasis on the fluorescein dye disappearance test. Moreover, it aims to review the full spectrum of therapeutic strategies—from conservative to surgical—along with their indications, effectiveness, and potential complications. The ultimate goal is to highlight the importance of early diagnosis and personalized treatment planning in order to ensure optimal outcomes and minimize the risk of serious complications in affected children.

## 2. Materials and Methods

To investigate the diagnostic methods, epidemiology, and management strategies associated with CNLDO in the pediatric population, a broad literature review was conducted using the PubMed, Scopus, and Web of Science databases. The search was carried out between November and December 2024, and included articles published from 2000 to 2024. The year 2000 was selected as a starting point to ensure that the analysis would encompass both foundational and contemporary medical practices, providing insight into the evolution of diagnostic and therapeutic approaches over more than two decades. Keywords and search strings were constructed using Boolean operators, and comprised terms such as: “congenital nasolacrimal duct obstruction”, “congenital tear duct obstruction”, “infant epiphora”, “lacrimal drainage disorders” and “neonatal dacryostenosis”. Articles qualified for inclusion if they were published in English in peer-reviewed journals, and if their primary focus was on the causes, clinical manifestations, diagnostic standards, or therapeutic approaches related to CNLDO in individuals aged 0–18 years. Additionally, studies offering valuable epidemiological data or insight into the evolution of clinical guidelines were prioritized. Articles were excluded if they were not available in English, were duplicates, appeared as conference abstracts, were animal experiments or in vitro studies, unless they contained contextual information relevant to human clinical care. After the removal of duplicate entries, 694 unique articles remained for initial title and abstract screening. Out of these, 120 full-text studies were selected for thorough evaluation. Following this process, 62 articles were ultimately included in the review based on relevance, methodological quality, population size, citation metrics, and recentness. Although this paper is structured as a narrative review and does not employ a systematic approach such as PRISMA (Preferred Reporting Items for Systematic reviews and Meta-Analyses), attention was paid to methodological transparency and comprehensiveness. Recognized limitations include the possibility of publication bias and inconsistent accessibility of full texts. Nonetheless, the goal was to synthesize a diverse and representative body of evidence that captures contemporary clinical perspectives on CNLDO.

## 3. Discussion

### 3.1. Etiopathogenesis and Epidemiology

#### 3.1.1. Etiopathogenesis

**Figure 1:**
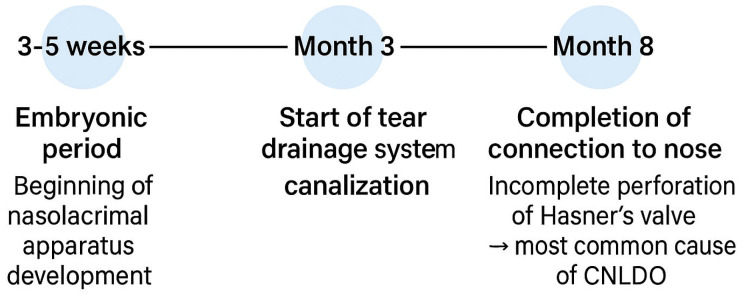
Development of the NLD during the gestational period.

**Figure 2:**
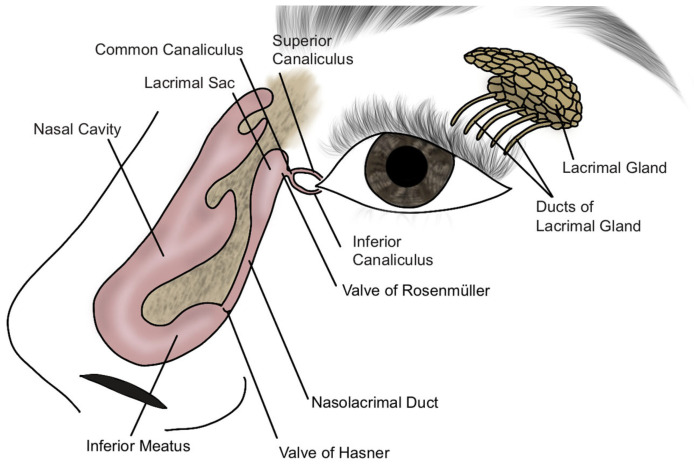
Diagram of the structure of the lacrimal organ.

The nasolacrimal apparatus develops during the embryonic period, between the third and fifth weeks of fetal life. The process of canalization of the tear drainage system begins in the third month of gestation, and the complete connection between the eyelid and nose is typically established by the eighth month of pregnancy [6]. Figure 1 presents the developmental stages of the nasolacrimal duct, while Figure 2 illustrates the schematic anatomy of the nasolacrimal system. CNLDO is the result of incomplete obstruction of the NLD during development. Obstruction can occur due to persistence of the embryonic membrane, abnormalities in the development of bony structures, or narrowing of the distal part of the NLD in the lower nasal passage [7]. The most common etiology is a mechanical blockage in the distal portion of the NLD, in proximity to Hasner’s valve, where the duct exits the nose. In less frequent instances, obstruction may develop in a more proximate location, such as the vicinity of the lacrimal sac, in proximity to the Rosenmüller valve [8].

#### 3.1.2. Epidemiology

CNLDO is the most prevalent congenital condition associated with tear outflow pathology. It has been observed in 6–20% of newborns [9]. A substantial proportion of these cases, approximately 80%, manifest as unilateral conditions [10]. The spontaneous resolution of the condition, in conjunction with the administration of conservative treatment, has been documented in 89% to 96% of children as early as the first year of life [11]. Research has demonstrated that among patients exhibiting severe CNLDO, 35% exhibit complete obstruction of the duct, 15% present with lacrimal point agenesis, 10% manifest congenital fistulas, and 5% display craniofacial bone defects [12,13]. Genetic predisposition, perinatal infection, exposure to radiation and certain drugs have been identified as risk factors for CNLDO. A higher incidence of the condition has been observed in premature infants compared to children born at full term [14].

CNLDO often co-occurs with other conditions. Children with Down syndrome, ectodermal dysplasia syndrome, cleft lip or palate, gill-eye-facial syndrome, CHARGE syndrome (which includes coloboma, heart defects, posterior nostril atresia, developmental delays, genital and ear anomalies), and Goldenhar syndrome are at increased risk of developing this disease [12]. CNLDO has been observed in up to 30% of individuals with Down syndrome [15].

### 3.2. Symptoms and Diagnosis

CNLDO typically emerges within the first weeks or months of life. In as many as 95% of children, symptoms are noticeable already in the first month of life The most common complaints reported by parents are tearing and/or the presence of a mucopurulent discharge [14]. Distal obstruction within the Hasner valve is more likely to result in mucopurulent discharge, while obstruction located closer to the lacrimal sac, in the Rosenmüller valve area, is more frequently associated with watery discharge. Furthermore, the manifestation of clumping of the eyelid skin has been observed. The presence of scabs on the eyelashes, accompanied by redness and abrasions on the eyelid skin resulting from incessant rubbing, has been documented [16].

Epiphora can cause visual impairments, such as blurred or distorted vision, which can hinder the emmetropization process. The period of greatest intensity for this process occurs between the third and ninth months of life, which coincides with the period when CNLDO symptoms reach their greatest intensity. The abnormal course of emmetropization can result in clinically significant hyperopia, affecting between 2% and 8% of children after infancy [17,18]. Children with CNLDO show an increased incidence of amblyopia, reaching approximately 5%. It is most commonly associated with anisometropia or strabismus, typically affecting the eye on the obstructed side, suggesting that chronic tearing may interfere with normal visual development [19].

This condition generally has a benign course; however, if untreated, recurrent conjunctivitis and lacrimal sac inflammation can develop. These conditions, in turn, can lead to recurrent eye infections, preconjunctivitis or orbital cellulitis, septicemia and even meningitis and brain abscess [20].

Prior to arriving at a diagnosis, it is imperative to rule out other potential etiologies of epiphora in infants, including epiblepharon (a congenital condition where a fold of skin causes the eyelashes to touch the ocular surface), congenital entropion, congenital glaucoma, keratitis, and uveitis. To differentiate between CNLDO and these other entities, use of fluorescein dye disappearance test (FDDT), visual macroscopy focused on the external examination or slit lamp examination is recommended. As an initial step in diagnostic evaluation, macroscopic examination of the eye region is performed. This includes assessment of the facial skeleton, presence of clefts or scars, hypertelorism, eyelid position, palpebral fissure width, and lid margins. Special attention is directed to the lacrimal sac area for signs of fistulas, swelling, or skin discoloration, which may indicate abnormalities in the lacrimal drainage system [6]. However the FDDT is regarded as the optimal diagnostic modality for identifying cases of tear duct obstruction, given its capacity to non-invasively and reliably diagnose such obstructions, exhibiting a sensitivity of 90% and a specificity of 100% [21,22]. FDDT is a procedure in which a drop containing fluorescein dye is applied to the patient’s eyes. To ensure the accuracy of the results, it is imperative that the patient refrain from touching their eyes and that tears flowing down the patient’s cheeks remain undisturbed. The evaluation is conducted after a period of five minutes. Under normal conditions, the dye should drain into the nasal cavity within this time, provided that the tear outflow pathways are unobstructed. In the event of an obstruction, the dye will remain in the eye in large amounts or will flow out through the lower eyelid onto the cheek [23].

While the diagnosis of CNLDO can often be made without the need for imaging tests, there are instances in which such tests are necessary to plan further treatment. Conditions such as craniofacial malformations or Down syndrome frequently co-occur with CNLDO. In such cases, computer tomography scans can be utilized to confirm bony occlusion of the NLD [21].

### 3.3. Treatment

**Figure 3:**
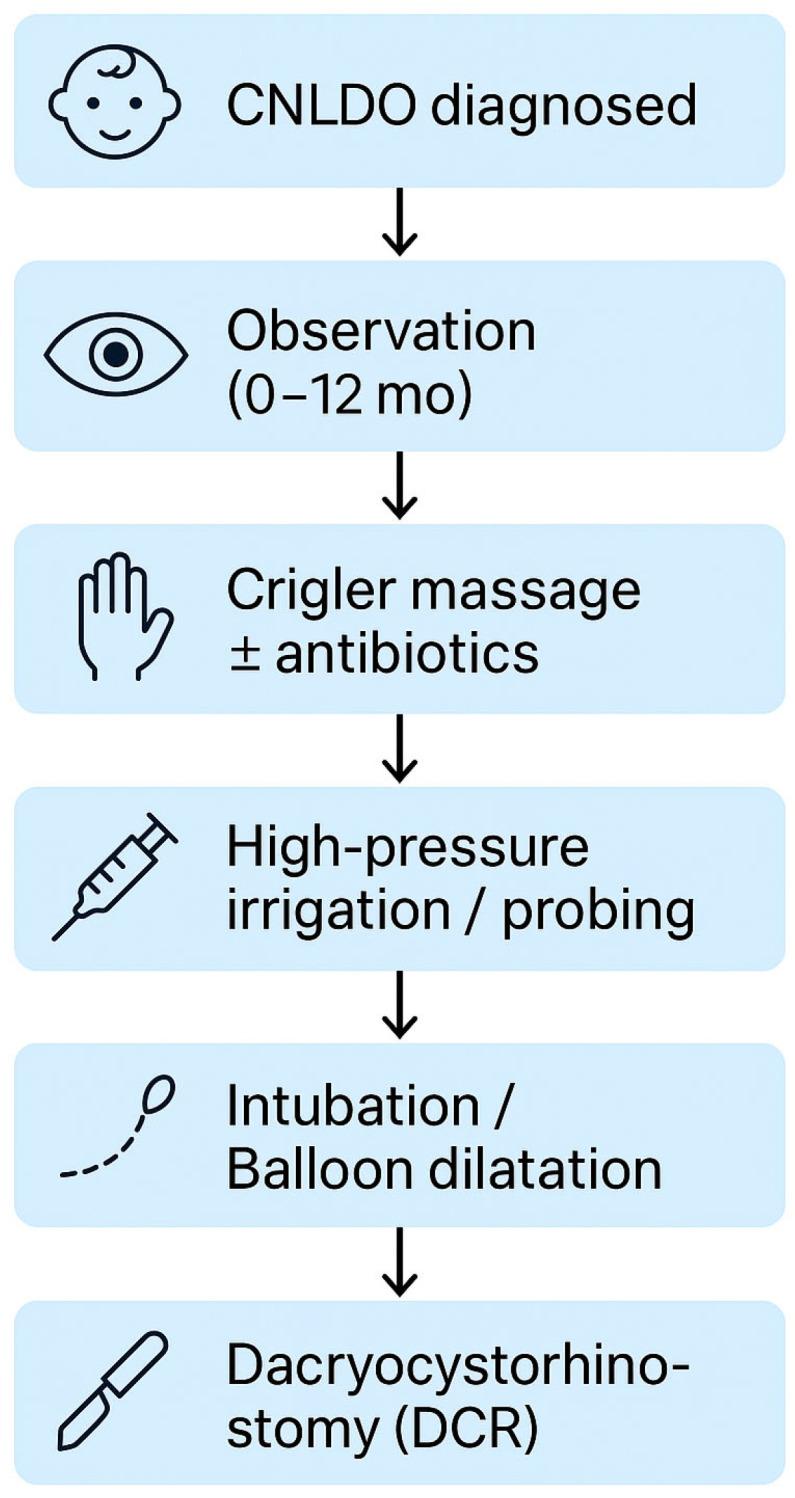
Stepwise Management of CNLDO.

The treatment of CNLDO, varies depending on factors such as age, symptom severity, and cause of obstruction, and includes multiple therapeutic options tailored to the individual case. Therapeutic approaches to CNLDO are broadly categorized into conservative and invasive measures. Conservative therapy, often the first line of management, is generally favored due to the high rate of spontaneous resolution observed in early infancy. Indications for transitioning to invasive interventions must be carefully weighed, taking into account other medical scenarios that necessitate escalation of care [6]. Despite the clinical relevance of this condition, no standardized international treatment guidelines for CNLDO have been established to date. Treatment strategies are typically applied in a stepwise manner, beginning with observation, followed by lacrimal sac massage (Crigler technique), and progressing to more interventional procedures such as high-pressure irrigation (HPI), probing, intubation, balloon dilation, and ultimately dacryocystorhinostomy (DCR) in refractory cases, as illustrated in Figure 3. A comparative overview of CNLDO treatments is presented in Table 1.

#### 3.3.1. Conservative Treatment

Conservative management is the cornerstone of initial treatment for CNLDO, given the high rate of spontaneous resolution in infants. Studies have shown that approximately 90% of cases resolve naturally within the first year of life, emphasizing the effectiveness of observation as a non-invasive first step in managing this condition [24]. The spontaneous resolution of CNLDO is primarily attributed to the gradual maturation of the NLD system and the natural opening of the valve of Hasner, supported by factors such as tear flow, blinking mechanics, and minor anatomical changes [25].

If spontaneous resolution does not take place or complications emerge during the observation period, conservative approaches such as NLD massage, also known as the Crigler technique, may be introduced as part of conservative management [26]. This method aims to promote the natural resolution of the obstruction by applying gentle pressure on the lacrimal sac to help clear blockages in the NLD [14]. As a continuation of the observation approach, the Crigler maneuver encourages ductal clearance and is particularly effective in younger infants [27]. The technique involves applying pressure near the inner corner of the eye along the lacrimal sac to alleviate the membranous obstruction, aiding in drainage and symptom relief. With a success rate of over 85% when performed properly, it plays a key role in non-invasive management and helps reduce the need for surgical treatment [28].

As per the guidelines, during the observation period, until the age of 4–5 months, gently wiping off eye discharge with wet, soft tissue is recommended, with antibacterial eye drops reserved for cases of severe or persistent discharge. Alongside massage, antibiotic eye drops, primarily fluoroquinolone derivatives, may be used, but only when there is associated conjunctivitis or a large amount of purulent discharge [41]. While these antibiotics can be helpful in controlling infection, frequent use may lead to the replacement of normal bacterial flora with antibiotic-resistant strains. Additionally, antibiotic drops can disrupt the surface of the eye, potentially causing further complications. Hygiene of the eyelashes and eyelids is also essential to prevent further irritation or infection [42].

The combination of observation, massage, and judicious use of antibiotics is typically the first-line approach for CNLDO due to its low risk and proven effectiveness in many cases [43]. This method is particularly appealing as it is easy to perform, does not require medical equipment and can be performed by parents or caregivers at home. Studies show that when this approach is not successful or symptoms continue beyond the first year, more invasive treatments, such as probing or surgery, may be necessary [32]. Thus, NLD massage is an essential part of the conservative treatment strategy, facilitating resolution before resorting to more invasive procedures [44].

#### 3.3.2. High Pressure Irrigation

HPI has gained recognition as an effective, minimally invasive procedure for treating CNLDO, especially when conservative methods like observation and massage have not succeeded [29]. Conducted under topical anesthesia in an outpatient setting, HPI involves the application of controlled pressure to flush the NLD, aiming to clear obstructions without surgical intervention. This approach bridges the gap between conservative management and invasive methods such as probing or intubation. HPI offers advantages like reduced patient discomfort and procedural risks, making it particularly appealing as a second-line treatment. Despite its minimally invasive nature and high success rate, HPI can be difficult to perform in younger children within outpatient settings, as their often limited cooperation, even under topical anesthesia, may compromise both the safety and effectiveness of the procedure. Nonetheless, it remains a valuable transitional option, offering a gentler intervention before considering more invasive surgical methods [45]

#### 3.3.3. Probing and Duct Intubation

When conservative management fails to resolve CNLDO, surgical intervention such as lacrimal duct probing is a commonly employed first-line treatment [30]. This procedure involves inserting a small, blunt probe or irrigation cannula into the lacrimal punctum, advancing it through the obstructed duct, and gently rupturing the membranous obstruction [33]. Irrigation with fluorescein-stained saline is often used to confirm patency and proper tear drainage. Probing can be performed in-office using local anesthesia or in the operating room under general anesthesia, with the latter being preferred for children older than 12 months to ensure their comfort and cooperation during the procedure [32,46].

The optimal timing for lacrimal duct probing remains a topic of debate among clinicians [33]. While some recommend early intervention between 6 and 10 months to minimize symptoms and avoid general anesthesia, others advocate for delaying the procedure until the child reaches 12 months [21]. Early probing before 6 months of age is recommended in cases of mucocele (a mucus-filled cyst that forms due to the obliteration of the excretory ducts of the glands), lacrimal sac abscess or significant dacryocystocele (a dilatation of the lacrimal sac caused by a blockage in the lacrimal system) with chronic purulent inflammation persisting despite conservative treatment [41]. In cases in which dacryocystocele occurs bilaterally and extends into the nasal cavity, it can lead to airway obstruction or swallowing difficulties, in which case lacrimal probing is advised [47]. After 6 months of age, probing is advised for frequent, recurrent infectious dacryocystitis, while late probing (between 8 and 12 months) is considered for persistent CNLDO without recurrent infections [41]. These recommendations are summarized in Table 2.

Delayed probing allows additional time for spontaneous resolution, which is common in younger infants, and facilitates a more controlled intervention under general anesthesia when needed. Early probing offers benefits such as reduced symptom duration, but these must be weighed against the natural resolution rates and potential complications associated with anesthesia in very young patients [48].

However, a review by Kashkouli et al. highlights that the success of initial probing decreases with age, not due to chronic infection or fibrosis, but because older children with unresolved epiphora may have a more complex underlying form of CNLDO [49]. This finding suggests that age-related complexity of CNLDO should be considered when determining the timing of probing.

For cases involving persistent or severe distal CNLDO, recurrent symptoms following initial probing, or when significant stenosis is observed, NLD intubation may be employed [34]. This technique involves the placement of a silicone stent within the nasolacrimal system after probing to maintain ductal patency. Although traditionally reserved as a secondary intervention after failed probing, nasolacrimal intubation has also been utilized as a primary treatment in selected cases, particularly due to the easier placement and removal associated with monocanalicular stents [14]. Silicone tube intubation is recommended for lacrimal canalicular stenosis regardless of patient age [41]. The stent is typically left in place for 2 to 6 months and is removed in the office during a follow-up visit. Intubation is particularly effective in preventing re-obstruction and has demonstrated success rates ranging from 90% to 96% for primary treatments and approximately 84% for secondary procedures following failed probing attempts [35]. Nasolacrimal silicone tube intubation has also shown higher success rates than repeat probing [36].

Endoscopy-assisted nasal probing, a recent advancement performed under general anesthesia, has improved the success rate of NLD probing while minimizing complications such as false passage formation. A nasal endoscope is gently inserted into the nasal cavity, typically beneath the inferior turbinate, allowing direct visualization of the inferior meatus and the distal end of the nasolacrimal duct. Topical decongestants and nasal packing are used to reduce mucosal swelling and widen the cavity for better access. After pack removal and aspiration, the nasal cavity is examined to detect any obstructions or anatomical variations. This direct visualization ensures accurate probe placement and enhances the safety and efficacy of the procedure [50,51].

A small proportion of children, approximately 9% to 13%, may require secondary procedures, including secondary probing, particularly in cases where the initial probing is insufficient [31].

Probing and intubation are both effective and minimally invasive options with high success rates for managing CNLDO [52]. A study suggests that treatment success does not significantly differ between early and late probing if performed before 16 months of age, allowing flexibility in clinical decision-making [33]. If symptoms persist or recur after probing, it is advised to repeat the procedure after approximately 4–6 weeks. In cases where two probing attempts fail, further imaging diagnostics and otolaryngology consultation are recommended, with management tailored to the specific pathology. If NLD stenosis persists despite treatment, intubation is advised, whereas in cases of complete CNLDO, dacryocystorhinostomy (DCR) is recommended [41]. The final decision on treatment should be made by the attending physician monitoring the patient.

Both lacrimal duct probing and silicone intubation are effective and commonly used treatments for CNLDO, with comparable success rates if performed before 16 months. Probing is typically the first-line intervention, while intubation is reserved for failures, recurrences, or complex obstructions. DCR is indicated in cases of persistent, complete NLD occlusion.

#### 3.3.4. Balloon Dilatation

Balloon catheter dilatation is an advanced intervention for the management of CNLDO, particularly indicated in cases refractory to probing or involving complex obstructions [53]. The procedure involves the insertion of a catheter with an inflatable balloon into the NLD, which is then dilated under controlled pressure to restore patency [54]. Performed under general anestheendosia, this technique is associated with high success rates and serves as an effective alternative to other approaches, including more invasive surgical methods, particularly for older children or those with persistent stenosis [55,56]

#### 3.3.5. Dacryocystorhinostomy

DCR is a surgical procedure reserved for cases of CNLDO that have failed other treatment modalities. The procedure creates a direct connection between the lacrimal sac and the nasal cavity, bypassing the obstructed NLD [6].

DCR can be performed via two main approaches:

- External DCR:
  - Involves a skin incision over the anterior lacrimal crest, preparation of the osteotomy and suturing of the lacrimal sac to the nasal mucosa to create a sac-nasal mucosal anastomosis;
  - Reported success rates are high, reaching approximately 96% in children;
- Endoscopic Endonasal DCR:
  - Performed without external incisions, minimizing visible scarring and preserving the medial canthus;
  - Nasal endoscopy facilitates identification and correction of intranasal abnormalities during surgery;
  - Success rates range from 82% to 94%, approaching those of external DCR [37];
  - This approach is preferred by many pediatric ophthalmologists due to its cosmetic and functional advantages [38];
  - It allows for precise visualization and management of the obstruction through nasal endoscopy, making it particularly suitable for complex or refractory cases.

DCR is typically considered the last-line surgical option, particularly for postsaccal bony atresia or complex obstructions [57]. The procedure is not recommended before the age of one year, as alternative techniques such as endoscopy may be sufficient for thin bony atresia. In cases of complete agenesis of the lacrimal ducts, conjunctivorhinostomy with a permanent bypass tube may be indicated. However, this surgery is generally postponed until facial bone growth is largely complete (10–12 years of age). Moderate-certainty evidence suggests that applying antimetabolites (e.g., mitomycin C) during DCR increases long-term functional and anatomical success rates [39]. While benefits are observed beyond six months post-surgery, their impact within the first six months remains inconclusive and adverse effects are minimal [40].

Revision surgeries may be required for failed primary DCR. Techniques include external DCR, endoscopic endonasal DCR and laser transcanalicular approaches. Revision outcomes are generally comparable regardless of the chosen technique, except when the primary surgery involved the laser transcanalicular approach, where external revision has shown superior outcomes [44].

DCR offers high success rates and is a critical intervention for refractory CNLDO. The choice between external and endoscopic approaches depends on the clinical context, patient age, and surgeon expertise. Delaying surgery until after one year of age is recommended unless alternative techniques are inadequate for the obstruction type [58]

#### 3.3.6. Other Approaches

In addition to standard probing, other specialized specialized approaches have been developed to manage complex or refractory cases of CNLDO. One such method is dacryoendoscopy (DE), a minimally invasive and effective tool for diagnosing and treating CNLDO, particularly in complex or persistent cases within the first year of life [8,59]. The procedure involves anesthetizing the patient, dilating the lacrimal punctum, and inserting an endoscope with saline irrigation to visualize and clear obstructions by puncturing or semi-blind probing when necessary [8]. DE offers superior direct visualization, enabling differentiation between obstruction types, including those caused by infection or anatomical anomalies, and guides appropriate treatment decisions ranging from probing to DCR. Early DE intervention shows high success rates with minimal complications and reliable long-term patency [8,60].

Further approaches include conjunctivorhinostomy, which is typically reserved for cases of complete agenesis of the NLD or severe, refractory obstructions where traditional DCR techniques fail. This technique involves creating a bypass between the conjunctiva and the nasal cavity, often with the insertion of a permanent bypass tube [61]. Additionally, laser-assisted procedures, such as laser transcanalicular DCR, offer a minimally invasive alternative to conventional DCR. These methods use laser technology to create an osteotomy through the lacrimal sac, and are particularly suitable for selecting patients with recurrent or complex obstructions [62].

Collectively, these advanced techniques are tailored to the severity and underlying etiology of the obstruction, providing customized and effective treatment strategies for the most challenging CNLDO cases.

## 4. Conclusions

CNLDO is an anatomical anomaly of the tear drainage system resulting from incomplete occlusion of NLD during prenatal development. The condition affects up to 20% of newborns. Most cases resolve spontaneously within the first year of life, due to the natural maturation of the nasolacrimal system. Symptoms include excessive tearing and the presence of mucous discharge, which can cause secondary complications such as eyelid skin sticking, recurrent conjunctivitis or inflammation of the tear sac. Diagnosis is primarily clinical, with FDT being the standard, and imaging reserved for complex cases. Initial treatment is conservative—observation, lacrimal sac massage and topical antibiotics. In refractory cases, probing, intubation, high-pressure irrigation, balloon dilation, or DCR may be required. The paper emphasizes the importance of early diagnosis and a personalized approach to therapy, which enables effective treatment and minimizes the risk of severe complications in patients with CNLDO.

## Figures and Tables

**Table 1:** Comparative Overview of CNLDO Treatment Methods.

Method	Target Age	Success Rate (%)	Invasiveness	Anesthesia	Remarks	Study, Year
Observation	0–12 months	~90%	None	Not applicable	High effectiveness in infants	Repka, 2018 [24]; Irfan, 2020 [25]
Crigler Massage	0–12 months	>85%	Low	No	Can be performed at home by parents	Bansal et al., 2021 [26]; Lekskul et al., 2022 [27]; Srivastava et al., 2023 [28]
High-Pressure Irrigation	>6 months	80–90%	Low	Topical	Quick outpatient procedure	Kashkouli, 2006 [29]
Probing	6–16 months	80–95%	Moderate	Depends on age	May require repetition; performed under general/local anesthesia	Örge et al., 2014 [30]; Petris et al., 2017 [31]; Świerczyńska et al., 2020 [32]; Farat et al., 2021 [33]
Intubation	>12 months	85–96%	Moderate	General	Effective in recurrent or resistant cases	Okumuş et al., 2016 [34]; Abushnein et al., 2024 [35]; Jafarizadeh et al., 2024 [36]
DCR * (external/endoscopic)	>1 year (preferred)	82–96%	High	General	Final-line treatment for complex or refractory obstructions	Heichel, 2024 [6]; Moreira et al., 2019 [37]; Cui et al., 2019 [38]; Freitag et al., 2023 [39]; Phelps et al., 2020 [40]

* dacryocystorhinostomy

**Table 2:** Timing and clinical indications for lacrimal duct probing in infants [41,47].

Age Range	Indications for Probing
0–6 months	- Mucocele - Lacrimal sac abscess - Significant dacryocystocele with chronic purulent discharge - Bilateral dacryocystocele witch airway obstruction
6–10 months	- Recurrent infectious dacryocystitis
8–12 months	- Persistent CNLDO without recurrent infections

## Data Availability

Not applicable.

## Abbreviations

The following abbreviations are used in this manuscript:

CNLDO	Congenital nasolacrimal duct obstruction
NLD	Nasolacrimal duct
FDDT	Fluorescein dye disappearance test
DCR	Dacryocystorhinostomy
DE	Dacryoendoscopy
